# Preoperative Breast Immune Prognostic Index as Prognostic Factor Predicts the Clinical Outcomes of Breast Cancer Patients Receiving Neoadjuvant Chemotherapy

**DOI:** 10.3389/fimmu.2022.831848

**Published:** 2022-03-07

**Authors:** Li Chen, Xiangyi Kong, Shaolong Huang, Zhaohui Su, Mengliu Zhu, Yi Fang, Lin Zhang, Xingrui Li, Jing Wang

**Affiliations:** ^1^ Department of Thyroid and Breast Surgery, Tongji Hospital, Tongji Medical College, Huazhong University of Science and Technology, Wuhan, China; ^2^ Department of Breast Surgical Oncology, National Cancer Center/National Clinical Research Center for Cancer/Cancer Hospital, Chinese Academy of Medical Sciences and Peking Union Medical College, Beijing, China; ^3^ Department of Thyroid & Breast, Burn and Plastic Surgery, Tongren City People’s Hospital, Tongren, China; ^4^ Center on Smart and Connected Health Technologies, Mays Cancer Center, School of Nursing, University of Texas (UT) Health San Antonio, San Antonio, TX, United States; ^5^ Melbourne School of Population and Global Health, The University of Melbourne, Melbourne, VIC, Australia; ^6^ Centre of Cancer Research, Victorian Comprehensive Cancer Centre, Melbourne VIC, Australia; ^7^ School of Population Medicine and Public Health, Chinese Academy of Medical Sciences and Peking Union Medical College, Beijing, China

**Keywords:** breast cancer, breast immune prognostic index, nomogram, neoadjuvant chemotherapy, survival

## Abstract

**Objective:**

This study aims at investigating the potential prognostic significance of the breast immune prognostic index (BIPI) in breast cancer patients who received neoadjuvant chemotherapy (NACT).

**Methods:**

The optimal cutoff value was calculated through the receiver operating characteristic curve (ROC). The correlations between BIPI and clinicopathologic characteristics were determined by the chi-square test or Fisher’s exact test. The Kaplan–Meier method was used to estimate the survival probability, and the log-rank test was used to analyze the differences in the survival probability among patients. The univariate and multivariate Cox proportional hazard regression model was used to screen the independent prognostic factors. A prognostic nomogram for disease-free survival (DFS) and overall survival (OS) was built on the basis of the multivariate analyses. Furthermore, the calibration curve and decision curve analysis (DCA) were used to assess the predictive performance of the nomogram.

**Results:**

All enrolled patients were split into three subgroups based on the BIPI score. The mean DFS and OS of the BIPI score 0 group and BIPI score 1 group were significantly longer than those of the BIPI score 2 group (42.02 vs. 38.61 vs. 26.01 months, 77.61 vs. 71.83 vs. 53.15 months; p < 0.05). Univariate and multivariate analyses indicated that BIPI was an independent prognostic factor for patients’ DFS and OS (DFS, hazard ratio (HR): 6.720, 95% confidence interval (CI): 1.629–27.717; OS, HR: 8.006, 95% CI: 1.638–39.119). A nomogram with a C-index of 0.873 (95% CI: 0.779–0.966) and 0.801 (95% CI: 0.702–0.901) had a favorable performance for predicting DFS and OS survival rates for clinical use by combining immune scores with other clinical features. The calibration curves at 1-, 3-, and 5-year survival suggested a good consistency between the predicted and actual DFS and OS probability. The DCA demonstrated that the constructed nomogram had better clinical predictive usefulness than only BIPI in predictive clinical applications of 5-year DFS and OS prognostic assessments.

**Conclusions:**

The patients with low BIPI score have better prognoses and longer DFS and OS. Furthermore, the BIPI-based nomogram may serve as a convenient prognostic tool for breast cancer and help in clinical decision-making.

## Introduction

Breast cancer (BC) is a fatal disease—it is the most common female malignancy and the primary cause of cancer-related death worldwide (1). Although the prognosis of breast cancer is relatively satisfactory in contrast to other tumors, such as gastrointestinal tumor and lung cancer, the survival outcome of patients with advanced breast cancer or with distant metastasis is still very poor (2). Moreover, more than half of breast cancer deaths are caused by distant metastasis (2). In the past, chemotherapy is the main treatment for advanced breast cancer or recurrent breast cancer (3). Over the past few decades, a great deal of molecular target drugs, for instance, monoclonal HER2-targeting antibodies (trastuzumab and pertuzumab) and antibody-drug conjugates (ADCs) (trastuzumab deruxtecan and trastuzumab emtansine), had been approved for the treatment of HER2-positive breast cancer or metastatic breast cancer (4, 5). Furthermore, immunotherapy (immune-checkpoint inhibitors) has been the focus of attention, and its effectiveness in the treatment of breast cancer has been reported (6, 7). The emergence and rise of these therapeutic agents have significantly improved the treatment of breast cancer.

Recently, some oncologists begin to focus on antitumor immune responses, which may become fundamental markers in cancer immunotherapy (8). Immune checkpoint inhibitors (ICIs), such as programmed cell death 1 (PD-1), programmed cell death ligand 1 (PD-L1), and cytotoxic T lymphocyte antigen 4 (CTLA-4), have indicated remarkable improvement in the prognosis for the treatment of dozens of cancers (9). However, mixed findings are also present in the immunotherapy literature, with the most noticeable one being that substantial heterogeneity in response is observed among different tumors (10). To address this issue, potential predictive biomarkers such as gene signatures and multi-omics have been used to further evaluate the prognosis of different tumors (11). However, obtaining and analyzing these biomarkers are often time-consuming, inconvenient, and expensive, which in turn could limit their clinical applications. Hence, it is necessary to develop effective and efficient indicators to evaluate the effect of immune status on the prognosis for breast cancer patients.

A systemic immune and inflammatory status in the body is of importance in cancer prognosis (12). The peripheral blood biomarkers representing inflammation and tumor burden have been increasingly studied in order to predict the treatment effect for breast cancer (13). Currently, some reports have shown that the neutrophil to lymphocyte ratio (NLR), monocyte to lymphocyte ratio (MLR), platelet to lymphocyte ratio (PLR), prognostic nutritional index (PNI), systemic immune inflammation index (SII), and systemic inflammation response index (SIRI) were used to reflect the patients’ immune and inflammatory status (14–19). Furthermore, a derived score composed of the white cell and neutrophil counts which are divided by absolute white cell count minus absolute neutrophil count (dNLR) is similar to NLR and can evaluate the prognosis of tumors (20). The baseline serum lactate dehydrogenase (LDH) level is also an independent prognostic factor for evaluating the survival outcomes in different cancer types, such as non-small cell lung cancer (NSCLC), metastatic melanoma, and colorectal cancer (21–23). Furthermore, the immune prognostic index (IPI) based on the LDH and the dNLR level can also help clinicians to examine and evaluate the prognosis in NSCLC (24). Moreover, the IPI stratified patients into poor, intermediate, and good prognostic groups to further improve the breast cancer diagnostic procedure (25). However, due to lack of research insights, whether the IPI is useful for the prognosis of breast cancer remains unclear, especially in breast cancer patients who received neoadjuvant chemotherapy (NACT). Therefore, to bridge the research gap, in the present study, we aim to gain insights into the clinical prognostic significance of the breast immune prognostic index (BIPI) as a useful prognostic factor in breast cancer patients undergoing NACT.

## Methods

### Ethics Approval and Consent to Participate

The present study was retrospectively conducted and approved by the institutional review board of the Cancer Hospital Chinese Academy of Medical Sciences in China. All processes performed in the study were conducted in accordance with the standards of the institutional research committee and with the declaration of 1964 Helsinki as well as its later amendments or comparable ethical standards. Individual patient information has been protected and not been shown.

### Study Population and Data Collection

We conducted a retrospective study of breast cancer undergoing NACT diagnosed and treated at the Cancer Hospital Chinese Academy of Medical Sciences between June 2009 and December 2015. Using the electronic medical records, we collected and searched the clinical and demographic data on every patient.

### Inclusion Criteria and Exclusion Criteria

Participants who met the following inclusion criteria were included in the study: 1) all enrolled breast patients who received NACT; 2) pathologically confirmed breast cancer, and underwent surgery after NACT; 3) no preoperative antitumor therapy or anti-infection treatments; 4) complete follow-up information and available clinical data; and 5) peripheral blood samples collected before treatment. The patients were excluded in the study if they have the following: 1) lack of clear and definite pathological diagnosis and medical history information; 2) with other malignant tumors except breast cancer or with distant metastasis; 3) suffer from autoimmune diseases or chronic inflammatory; and 4) with history of blood transfusion before treatment.

### Calculation of the Breast Immune Prognostic Index

The BIPI was an indicator that combined the LDH level and the dNLR level. The dNLR was defined as neutrophil count/(white blood cell count – neutrophil count). The dNLR had been recently identified as a prognostic factor of immune checkpoint inhibitor therapy (26). The optimal cutoff values of LDH and dNLR were assessed by the ROC curve (**Figure S1**). The optimal cutoff values of LDH and dNLR were 203.5 U/l (range: 105–715 U/l) and 1.67 (range: 0.07–4.36), respectively. Moreover, all patients were assigned to three groups: 1) BIPI score 0 (Good): LDH < 203.5 U/l and dNLR < 1.67; 2) BIPI score 1 (Intermediate): LDH ≥203.5 U/l and dNLR < 1.67, or LDH < 203.5 U/l and dNLR ≥ 1.67; and 3) BIPI score 2 (Poor): LDH ≥203.5 U/l and dNLR ≥ 1.67. According to the BIPI score, 43 (41.3%), 46 (44.2%), and 15 14.4%) breast cancer patients were classified into the BIPI score 0 group, BIPI score 1 group, and BIPI score 2 group, respectively.

### Follow-Up

All enrolled patients had routine inpatient, outpatient, and/or telephone follow-up after operation. Follow-up evaluations were performed every 3 months for the first to the second year, every 6 months for the third to the fifth year, and then yearly thereafter. Disease-free survival (DFS) was defined as the time lapsed from surgery to progression with regard to the distant disease metastasis, death from any cause, or last follow-up. Overall survival (OS) was defined as the time lapsed from surgery to the date of death from any cause or last follow-up.

### Statistical Analysis

The baseline characteristics data were presented as absolute value and percentage (%), compared between groups using the chi-square test or Fisher’s exact test. The optimal cutoff value was calculated using the receiver operating characteristic curve (ROC). The Kaplan–Meier method was used to estimate the survival probability, and the log-rank test was used to compare survival distributions of the individual index level. The univariate and multivariate Cox proportional hazard regression model was used to evaluate the independent prognostic factors. The hazard ratios (HRs) and 95% confidence intervals (CIs) were performed to evaluate the association between the clinicopathological data. The prognostic nomogram for DFS and OS was established on the multivariate analyses. The calibration curve and decision curve analysis (DCA) were further used to assess the predictive performance. All statistical analyses were performed using the SPSS software (version 17.0; SPSS Inc., Chicago, IL, USA), GraphPad Prism software (version 8.0; GraphPad Inc., La Jolla, CA, USA), and R (version 3.6.0; Vienna, Austria. URL: http://www.R-project.org/). Alpha was set at the 0.05 level, and a two-tailed p < 0.05 indicated statistical significance.

## Results

### Patients’ General Characteristics in the Study

A total of 104 breast cancer patients who received NACT were included in the present study. The median age was 46 years (range from 27 to 73 years). On the basis of the eighth edition of the TNM classification, 3 (2.9%), 39 (37.5%), and 62 (59.6%) breast cancer patients before treatment were classified as stages I, II, and III, respectively. After operation, 2 (1.9%), 16 (15.4%), 38 (36.5%), and 48 (46.2%) breast cancer patients were classified as stages Tis/T0, I, II, and III, respectively. In terms of ABO blood type, A type was 28 cases (26.9%), B type was 34 cases (32.7%), O type was 28 cases (26.9%), and AB type was 14 cases (13.5%). The PD-1 and PD-L1 protein expressions in tumor cells by immunohistochemistry (IHC) assay were 38 cases with PD-1 low expression, 66 cases with PD-1 high expression, 61 cases with PD-L1 low expression, and 43 cases with PD-L1 high expression, respectively. BIPI was associated with type of surgery (p = 0.032). The clinical characteristics are summarized in **Table 1**.

**Table 1:** Patients’ characteristics for all patients in accordance with breast immune prognostic index (BIPI).

n	Level	BIPI score 0	BIPI score 1	BIPI score 2	p
		43	46	15	
Age (%)	<46	22 (51.2)	20 (43.5)	6 (40.0)	0.672
	≥46	21 (48.8)	26 (56.5)	9 (60.0)	
BMI (%)	<23.63	24 (55.8)	22 (47.8)	3 (20.0)	0.057
	≥23.63	19 (44.2)	24 (52.2)	12 (80.0)	
Family history (%)	No	35 (81.4)	34 (73.9)	11 (73.3)	0.661
	Yes	8 (18.6)	12 (26.1)	4 (26.7)	
Menarche age (%)	<14	15 (34.9)	18 (39.1)	6 (40.0)	0.897
	≥14	28 (65.1)	28 (60.9)	9 (60.0)	
Menopause (%)	No	27 (62.8)	28 (60.9)	9 (60.0)	0.974
	Yes	16 (37.2)	18 (39.1)	6 (40.0)	
ABO blood type (%)	A	10 (23.3)	15 (32.6)	3 (20.0)	0.927
	B	14 (32.6)	15 (32.6)	5 (33.3)	
	O	13 (30.2)	10 (21.7)	5 (33.3)	
	AB	6 (14.0)	6 (13.0)	2 (13.3)	
Tumor site (%)	Right	17 (39.5)	23 (50.0)	5 (33.3)	0.428
	Left	26 (60.5)	23 (50.0)	10 (66.7)	
Clinical T stage (%)	T1	9 (20.9)	4 (8.7)	2 (13.3)	0.422
	T2	23 (53.5)	26 (56.5)	8 (53.3)	
	T3	7 (16.3)	6 (13.0)	1 (6.7)	
	T4	4 (9.3)	10 (21.7)	4 (26.7)	
Clinical N stage (%)	N0	6 (14.0)	10 (21.7)	0 (0.0)	0.219
	N1	16 (37.2)	16 (34.8)	3 (20.0)	
	N2	16 (37.2)	13 (28.3)	8 (53.3)	
	N3	5 (11.6)	7 (15.2)	4 (26.7)	
Clinical TNM stage (%)	I	2 (4.7)	1 (2.2)	0 (0.0)	0.455
	II	17 (39.5)	19 (41.3)	3 (20.0)	
	III	24 (55.8)	26 (56.5)	12 (80.0)	
Operative time (%)	<90	15 (34.9)	25 (54.3)	9 (60.0)	0.103
	≥90	28 (65.1)	21 (45.7)	6 (40.0)	
Type of surgery (%)	Mastectomy	32 (74.4)	41 (89.1)	15 (100.0)	0.032
	Breast-conserving surgery	11 (25.6)	5 (10.9)	0 (0.0)	
Pathological tumor size (%)	≤2 cm	21 (48.8)	19 (41.3)	5 (33.3)	0.713
	>2 and <5 cm	20 (46.5)	24 (52.2)	8 (53.3)	
	≥5 cm	2 (4.7)	3 (6.5)	2 (13.3)	
Histologic grade (%)	I	4 (9.3)	2 (4.3)	0 (0.0)	0.382
	II	25 (58.1)	32 (69.6)	8 (53.3)	
	III	14 (32.6)	12 (26.1)	7 (46.7)	
Pathological T stage (%)	Tis/T0	2 (4.7)	2 (4.3)	0 (0.0)	0.523
	T1	20 (46.5)	16 (34.8)	5 (33.3)	
	T2	20 (46.5)	21 (45.7)	8 (53.3)	
	T3	0 (0.0)	2 (4.3)	0 (0.0)	
	T4	1 (2.3)	5 (10.9)	2 (13.3)	
Pathological N stage (%)	N0	13 (30.2)	14 (30.4)	4 (26.7)	0.893
	N1	12 (27.9)	12 (26.1)	3 (20.0)	
	N2	9 (20.9)	8 (17.4)	2 (13.3)	
	N3	9 (20.9)	12 (26.1)	6 (40.0)	
Pathological TNM stage (%)	Tis/T0	1 (2.3)	1 (2.2)	0 (0.0)	0.952
	I	7 (16.3)	6 (13.0)	3 (20.0)	
	II	17 (39.5)	17 (37.0)	4 (26.7)	
	III	18 (41.9)	22 (47.8)	8 (53.3)	
Total lymph node (TLN) (%)	<24	21 (48.8)	26 (56.5)	6 (40.0)	0.505
	≥24	22 (51.2)	20 (43.5)	9 (60.0)	
Positive lymph node (PLN) (%)	<2	17 (39.5)	20 (43.5)	6 (40.0)	0.925
	≥2	26 (60.5)	26 (56.5)	9 (60.0)	
Lymph vessel invasion (%)	Negative	28 (65.1)	28 (60.9)	6 (40.0)	0.227
	Positive	15 (34.9)	18 (39.1)	9 (60.0)	
Neural invasion (%)	Negative	34 (79.1)	35 (76.1)	12 (80.0)	0.923
	Positive	9 (20.9)	11 (23.9)	3 (20.0)	
Postoperative chemotherapy (%)	No	13 (30.2)	13 (28.3)	4 (26.7)	0.960
	Yes	30 (69.8)	33 (71.7)	11 (73.3)	
Postoperative radiotherapy (%)	No	9 (20.9)	12 (26.1)	4 (26.7)	0.823
	Yes	34 (79.1)	34 (73.9)	11 (73.3)	
Postoperative endocrine therapy (%)	No	20 (46.5)	17 (37.0)	7 (46.7)	0.616
	Yes	23 (53.5)	29 (63.0)	8 (53.3)	
Postoperative targeted therapy (%)	No	31 (72.1)	30 (65.2)	11 (73.3)	0.729
	Yes	12 (27.9)	16 (34.8)	4 (26.7)	
PD1 (%)	Low expression	12 (27.9)	18 (39.1)	8 (53.3)	0.188
	High expression	31 (72.1)	28 (60.9)	7 (46.7)	
PDL1 (%)	Low expression	25 (58.1)	28 (60.9)	8 (53.3)	0.872
	High expression	18 (41.9)	18 (39.1)	7 (46.7)	

### Association Between BIPI and the Patients’ Pathology Parameters in the Study

There were 8 patients (7.7%) with Luminal A type, 14 patients (13.5%) with Luminal B HER2 (+) type, 35 patients (33.7%) with Luminal B HER2 (-) type, 15 patients (14.4%) with HER2-enriched type, and 32 patients (30.8%) with triple-negative type before NACT. Moreover, 17 patients (16.3%) with Luminal A type, 9 patients (8.7%) with Luminal B HER2 (+) type, 23 patients (22.1%) with Luminal B HER2 (-), 18 patients (17.3%) with HER2-enriched type, and 37 patients (35.6%) with triple-negative type after operation. BIPI was associated with P53 (p = 0.010). Detailed information is shown in **Table 2**.

**Table 2:** Patients’ pathology parameters for all patients in accordance with breast immune prognostic index (BIPI).

n	Level	BIPI score 0	BIPI score 1	BIPI score 2	p
		43	46	15	
**Core needle biopsy**					
Molecular subtype (%)	Luminal A	1 (2.3)	6 (13.0)	1 (6.7)	0.838
	Luminal B HER2+	7 (16.3)	5 (10.9)	2 (13.3)	
	Luminal B HER2-	14 (32.6)	16 (34.8)	5 (33.3)	
	HER2 enriched	7 (16.3)	6 (13.0)	2 (13.3)	
	Triple negative	14 (32.6)	13 (28.3)	5 (33.3)	
ER (%)	Negative	21 (48.8)	17 (37.0)	5 (33.3)	0.415
	Positive	22 (51.2)	29 (63.0)	10 (66.7)	
PR (%)	Negative	20 (46.5)	16 (34.8)	6 (40.0)	0.530
	Positive	23 (53.5)	30 (65.2)	9 (60.0)	
HER2 (%)	Negative	31 (72.1)	35 (76.1)	10 (66.7)	0.761
	Positive	12 (27.9)	11 (23.9)	5 (33.3)	
Ki67 (%)	Negative	6 (14.0)	13 (28.3)	1 (6.7)	0.095
	Positive	37 (86.0)	33 (71.7)	14 (93.3)	
**Postoperative pathology**					
Molecular subtype (%)	Luminal A	9 (20.9)	8 (17.4)	0 (0.0)	0.766
	Luminal B HER2+	3 (7.0)	5 (10.9)	1 (6.7)	
	Luminal B HER2-	8 (18.6)	10 (21.7)	5 (33.3)	
	HER2 enriched	8 (18.6)	7 (15.2)	3 (20.0)	
	Triple negative	15 (34.9)	16 (34.8)	6 (40.0)	
ER (%)^#^	Negative	23 (53.5)	21 (45.7)	4 (26.7)	0.199
	Positive	20 (46.5)	25 (54.3)	11 (73.3)	
PR (%)^#^	Negative	22 (51.2)	21 (45.7)	7 (46.7)	0.867
	Positive	21 (48.8)	25 (54.3)	8 (53.3)	
HER2 (%)^#^	Negative	33 (76.7)	36 (78.3)	11 (73.3)	0.925
	Positive	10 (23.3)	10 (21.7)	4 (26.7)	
Ki67 (%)	Negative	17 (39.5)	16 (34.8)	3 (20.0)	0.391
	Positive	26 (60.5)	30 (65.2)	12 (80.0)	
AR (%)^#^	Negative	37 (86.0)	40 (87.0)	14 (93.3)	0.755
	Positive	6 (14.0)	6 (13.0)	1 (6.7)	
CK5/6 (%)	Negative	34 (79.1)	34 (73.9)	7 (46.7)	0.051
	Positive	9 (20.9)	12 (26.1)	8 (53.3)	
E-cad (%)^#^	Negative	8 (18.6)	14 (30.4)	2 (13.3)	0.261
	Positive	35 (81.4)	32 (69.6)	13 (86.7)	
EGFR (%)^#^	Negative	23 (53.5)	28 (60.9)	6 (40.0)	0.360
	Positive	20 (46.5)	18 (39.1)	9 (60.0)	
P53 (%)	Negative	20 (46.5)	23 (50.0)	1 (6.7)	0.010
	Positive	23 (53.5)	23 (50.0)	14 (93.3)	
TOP2A (%)^#^	Negative	8 (18.6)	14 (30.4)	1 (6.7)	0.120
	Positive	35 (81.4)	32 (69.6)	14 (93.3)	
Lymph vessel invasion (%)	Negative	28 (65.1)	28 (60.9)	6 (40.0)	0.227
	Positive	15 (34.9)	18 (39.1)	9 (60.0)	
Neural invasion (%)	Negative	34 (79.1)	35 (76.1)	12 (80.0)	0.923
	Positive	9 (20.9)	11 (23.9)	3 (20.0)	

^#^ER, estrogen receptor; PR, progesterone receptor; HER2, human epidermal growth factor receptor 2; AR, androgen receptor; E-cad, E-cadherin; EGFR, epidermal growth factor receptor; TOP2A, topoisomerase II-α.

### Association Between BIPI and the Patients’ Chemotherapy in the Study

Based on the RECIST guidelines, there were 60 patients (57.7%) with partial responses (PRs), 43 patients (41.3%) with stable disease (SD), and one patient (1.0%) with progressive disease (PD) after two chemotherapy cycles. According to Miller–Payne grade (MPG), there were 9 cases (8.7%) with MPG 1, 42 cases (40.4%) with MPG 2, 48 cases (46.2%) with MPG 3, one case (1.0%) with MPG 4, and 4 cases (3.8%) with MPG 5. However, no significant correlations between BIPI and the patients’ chemotherapy were found (p > 0.05). Detailed information is shown in **Table 3**.

**Table 3:** Patients’ chemotherapy for all patients in accordance with breast immune prognostic index (BIPI).

n	Level	BIPI score 0	BIPI score 1	BIPI score 2	p
		43	46	15	
Neo-chemotherapy regimen (%)^#^	AC/ACF	2 (4.7)	1 (2.2)	1 (6.7)	0.615
	CT/ACT	5 (11.6)	2 (4.3)	3 (20.0)	
	AT	22 (51.2)	23 (50.0)	8 (53.3)	
	TP	7 (16.3)	12 (26.1)	2 (13.3)	
	Others	7 (16.3)	8 (17.4)	1 (6.7)	
Neo-chemotherapy times (%)	<6	17 (39.5)	14 (30.4)	3 (20.0)	0.347
	≥6	26 (60.5)	32 (69.6)	12 (80.0)	
Response (%)	PR	26 (60.5)	25 (54.3)	9 (60.0)	0.741
	SD	16 (37.2)	21 (45.7)	6 (40.0)	
	PD	1 (2.3)	0 (0.0)	0 (0.0)	
MPG (%)	1	4 (9.3)	3 (6.5)	2 (13.3)	0.756
	2	20 (46.5)	15 (32.6)	7 (46.7)	
	3	17 (39.5)	25 (54.3)	6 (40.0)	
	4	0 (0.0)	1 (2.2)	0 (0.0)	
	5	2 (4.7)	2 (4.3)	0 (0.0)	
Postoperative chemotherapy (%)	No	13 (30.2)	13 (28.3)	4 (26.7)	0.960
	Yes	30 (69.8)	33 (71.7)	11 (73.3)	
Postoperative chemotherapy regimen (%)	AC/ACF	3 (7.0)	6 (13.0)	0 (0.0)	0.847
	CT/ACT	2 (4.7)	3 (6.5)	1 (6.7)	
	AT	4 (9.3)	3 (6.5)	2 (13.3)	
	TP	8 (18.6)	8 (17.4)	1 (6.7)	
	Others	13 (30.2)	13 (28.3)	7 (46.7)	
	No	13 (30.2)	13 (28.3)	4 (26.7)	
Postoperative chemotherapy times (%)	<4	18 (41.9)	22 (47.8)	8 (53.3)	0.711
	≥4	25 (58.1)	24 (52.2)	7 (46.7)	

^#^Neo-chemotherapy regimen A, anthracyclines; C, cyclophosphamide; F, 5-fluorouracil; T, taxol; P, platinum compounds.

### Association Between BIPI and the Patients’ Side Effects of Chemotherapy in the Study

The hematologic reactions and gastrointestinal reactions myelosuppression and hepatic dysfunction were the common adverse events (AEs) among the NACT treatment. Nevertheless, no significant correlations between BIPI and side effects of chemotherapy were found (p > 0.05). Detailed information is shown in **Table 4**.

**Table 4:** Patients’ side effects of chemotherapy for all patients in accordance with breast immune prognostic index (BIPI).

n	Level	BIPI score 0	BIPI score 1	BIPI score 2	p
		43	46	15	
Decreased appetite (%)	No	6 (14.0)	10 (21.7)	1 (6.7)	0.335
	Yes	37 (86.0)	36 (78.3)	14 (93.3)	
Nausea (%)	No	5 (11.6)	6 (13.0)	0 (0.0)	0.346
	Yes	38 (88.4)	40 (87.0)	15 (100.0)	
Vomiting (%)	No	20 (46.5)	24 (52.2)	6 (40.0)	0.690
	Yes	23 (53.5)	22 (47.8)	9 (60.0)	
Diarrhea (%)	No	40 (93.0)	43 (93.5)	14 (93.3)	0.996
	Yes	3 (7.0)	3 (6.5)	1 (6.7)	
Mouth ulcers (%)	No	43 (100.0)	44 (95.7)	15 (100.0)	0.276
	Yes	0 (0.0)	2 (4.3)	0 (0.0)	
Alopecia (%)	No	20 (46.5)	24 (52.2)	4 (26.7)	0.227
	Yes	23 (53.5)	22 (47.8)	11 (73.3)	
Peripheral neurotoxicity (%)	No	37 (86.0)	35 (76.1)	15 (100.0)	0.081
	Yes	6 (14.0)	11 (23.9)	0 (0.0)	
Anemia (%)	Grade 0	23 (53.5)	27 (58.7)	5 (33.3)	0.231
	Grades 1–2	20 (46.5)	19 (41.3)	10 (66.7)	
Leukopenia (%)	Grade 0	11 (25.6)	10 (21.7)	3 (20.0)	0.581
	Grades 1–2	20 (46.5)	28 (60.9)	7 (46.7)	
	Grades 3–4	12 (27.9)	8 (17.4)	5 (33.3)	
Neutropenia (%)	Grade 0	9 (20.9)	7 (15.2)	4 (26.7)	0.582
	Grades 1–2	14 (32.6)	22 (47.8)	5 (33.3)	
	Grades 3–4	20 (46.5)	17 (37.0)	6 (40.0)	
Thrombocytopenia (%)	Grade 0	34 (79.1)	35 (76.1)	10 (66.7)	0.626
	Grades 1–2	9 (20.9)	11 (23.9)	5 (33.3)	
Gastrointestinal reaction (%)	Grade 0	5 (11.6)	6 (13.0)	1 (6.7)	0.756
	Grades 1–2	37 (86.0)	40 (87.0)	14 (93.3)	
	Grades 3–4	1 (2.3)	0 (0.0)	0 (0.0)	
Myelosuppression (%)	Grade 0	6 (14.0)	7 (15.2)	2 (13.3)	0.654
	Grades 1–2	10 (23.3)	17 (37.0)	4 (26.7)	
	Grades 3–4	27 (62.8)	22 (47.8)	9 (60.0)	
Hepatic dysfunction (%)	Grade 0	30 (69.8)	30 (65.2)	6 (40.0)	0.113
	Grades 1–2	13 (30.2)	16 (34.8)	9 (60.0)	

### Univariate and Multivariate Analyses for DFS and OS

The univariate analysis indicated that LDH, dNLR, BIPI, PD-L1, ABO blood type, pathological N stage, total lymph node (TLN), PR, Ki67, CK5/6, E-cadherin (E-cad), postoperative chemotherapy, postoperative endocrine therapy, and postoperative targeted therapy were associated with the prognosis of breast cancer patients for DFS; however, the multivariate analysis found that only LDH, BIPI, PD-L1, ABO blood type, PR, E-cad, postoperative chemotherapy, postoperative endocrine therapy, and postoperative targeted therapy were the independent prognostic factors for DFS (**Table 5**). Moreover, the results were displayed using forest plots and are shown in **Figure S2A**.

**Table 5:** Univariate and multivariate cox proportional hazard regression model for disease-free survival (DFS) and overall survival (OS).

Parameters	Level	DFS			p value	OS			p value
		Univariate analysis	p value	Multivariate analysis		Univariate analysis	p value	Multivariate analysis	
		Hazard ratio (95% CI)		Hazard ratio (95% CI)		Hazard ratio (95% CI)		Hazard ratio (95% CI)	
LDH	<203.5	1 (reference)	0.000	1 (reference)	0.000	1 (reference)	0.000	1 (reference)	0.000
	≥203.5	7.698 (2.548–23.257)		2.420 (1.490–3.932)		9.449 (2.848–31.351)		4.146 (2.437–7.054)	
dNLR	<1.67	1 (reference)	0.003			1 (reference)	0.012		
	≥1.67	6.477 (1.922–21.829)				5.471 (1.444–20.733)			
BIPI	Good	1 (reference)	0.026	1 (reference)	0.008	1 (reference)	0.018	1 (reference)	0.010
	Intermediate + Poor	5.110 (1.218–21.434)		6.720 (1.629–27.717)		6.773 (1.394–32.908)		8.006 (1.638–39.119)	
PD-1	Low expression	1 (reference)	0.101			1 (reference)	0.698		
	High expression	1.433 (0.933–2.201)				1.090 (0.707–1.680)			
PD-L1	Low expression	1 (reference)	0.027	1 (reference)	0.013	1 (reference)	0.007	1 (reference)	0.000
	High expression	0.566 (0.341–0.937)		0.605 (0.407–0.898)		0.461 (0.262–0.811)		0.420 (0.280–0.630)	
Age	<46	1 (reference)	0.880			1 (reference)	0.562		
	≥46	1.077 (0.413–2.808)				0.734 (0.257–2.091)			
ABO blood type	A+B	1 (reference)	0.026	1 (reference)	0.002	1 (reference)	0.395		
	O+AB	2.265 (1.104–4.647)		1.880 (1.269–2.787)		1.337 (0.684–2.615)			
Menopause	No	1 (reference)	0.858			1 (reference)	0.413		
	Yes	1.095 (0.404–2.967)				1.540 (0.548–4.326)			
White blood cell	<5.92	1 (reference)	0.929			1 (reference)	0.184		
	≥5.92	1.038 (0.454–2.376)				1.822 (0.752–4.415)			
Neutrophils	<3.66	1 (reference)	0.251			1 (reference)	0.035	1 (reference)	0.041
	≥3.66	0.612 (0.265–1.414)				0.359 (0.139–0.929)		0.481 (0.239–0.970)	
Lymphocyte	<1.75	1 (reference)	0.449			1 (reference)	0.043		
	≥1.75	0.764 (0.380–1.534)				0.515 (0.270–0.979)			
Monocyte	<0.37	1 (reference)	0.082			1 (reference)	0.015		
	≥0.37	1.965 (0.917–4.211)				2.673 (1.210–5.907)			
Tumor site	Right	1 (reference)	0.079			1 (reference)	0.001		
	Left	1.686 (0.942–3.019)				2.794 (1.520–5.136)			
Clinical T stage	T1	1 (reference)	0.429			1 (reference)	0.973		
	T2+T3+T4	1.407 (0.604–3.277)				0.985 (0.412–2.353)			
Clinical N stage	N0	1 (reference)	0.702			1 (reference)	0.091		
	N1+N2+N3	0.772 (0.206–2.897)				0.289 (0.069–1.216)			
Clinical TNM stage	I	1 (reference)	0.201			1 (reference)	0.978		
	II+III	0.173 (0.012–2.553)				1.045 (0.044–24.923)			
Response	PR	1 (reference)	0.466			1 (reference)	0.681		
	SD+PD	0.820 (0.480–1.399)				0.883 (0.489–1.595)			
MPG^#^	1+2	1 (reference)	0.747			1 (reference)	0.091		
	3+4+5	0.909 (0.510–1.621)				1.724 (0.917–3.242)			
Type of surgery	Mastectomy	1 (reference)	0.590			1 (reference)	0.301		
	Breast-conserving surgery	1.227 (0.583–2.583)				0.660 (0.300–1.450)			
Pathological tumor size	≤2 cm	1 (reference)	0.476			1 (reference)	0.365		
	>2 cm	1.745 (0.378–8.048)				0.461 (0.086–2.462)			
Histologic grade	I	1 (reference)	0.246			1 (reference)	0.836		
	II+III	2.759 (0.497–15.334)				0.821 (0.127–5.321)			
Pathological T stage	T1	1 (reference)	0.695			1 (reference)	0.183		
	T2+T3+T4	0.710 (0.128–3.932)				3.439 (0.558–21.192)			
Pathological N stage	N0	1 (reference)	0.045			1 (reference)	0.050		
	N1+N2+N3	4.415 (1.031–18.908)				4.344 (1.001–18.851)			
Pathological TNM stage	Tis/T0+I	1 (reference)	0.217			1 (reference)	0.004		
	II+III	2.557 (0.576–11.355)				12.298 (2.264–66.788)			
TLN^#^	<24	1 (reference)	0.036			1 (reference)	0.289		
	≥24	1.893 (1.042–3.439)				0.708 (0.374–1.340)			
PLN^#^	<2	1 (reference)	0.179			1 (reference)	0.006	1 (reference)	0.000
	≥2	0.555 (0.235–1.310)				3.566 (1.440–8.830)		3.352(1.987–5.653)	
Postoperative pathology									
Molecular subtype	Luminal A/B HER2+/B HER2-	1 (reference)	0.477			1 (reference)	0.258		
	HER2 enriched/triple negative	0.656 (0.205–2.100)				2.078 (0.585–7.374)			
ER^#^	Negative	1 (reference)	0.053			1 (reference)	0.389		
	Positive	0.247 (0.060–1.017)				1.969 (0.422–9.190)			
PR^#^	Negative	1 (reference)	0.000	1 (reference)	0.000	1 (reference)	0.000	1 (reference)	0.000
	Positive	10.383 (3.274–32.921)		3.776 (2.256–6.319)		29.838 (9.348–95.236)		4.852 (2.729–8.625)	
HER2^#^	Negative	1 (reference)	0.109			1 (reference)	0.051		
	Positive	0.486 (0.201–1.173)				0.434 (0.188–1.003)			
Ki67	Negative	1 (reference)	0.030			1 (reference)	0.107		
	Positive	2.125 (1.075–4.201)				1.771 (0.884–3.549)			
AR^#^	Negative	1 (reference)	0.690			1 (reference)	0.154		
	Positive	1.204 (0.484–2.995)				0.460 (0.158–1.339)			
CK5/6	Negative	1 (reference)	0.029			1 (reference)	0.000		
	Positive	0.353 (0.138–0.900)				0.115 (0.041–0.326)			
E-cad^#^	Negative	1 (reference)	0.010	1 (reference)	0.002	1 (reference)	0.005	1 (reference)	0.000
	Positive	2.593 (1.260–5.339)		2.103 (1.305–3.387)		3.224 (1.435–7.246)		2.778 (1.634–4.724)	
EGFR^#^	Negative	1 (reference)	0.522			1 (reference)	0.005		
	Positive	0.695 (0.228–2.121)				4.940 (1.607–15.187)			
P53	Negative	1 (reference)	0.460			1 (reference)	0.255		
	Positive	1.324 (0.629–2.789)				1.584 (0.718–3.499)			
TOP2A^#^	Negative	1 (reference)	0.744			1 (reference)	0.571		
	Positive	0.871 (0.380–1.998)				1.341 (0.486–3.703)			
Lymph vessel invasion	Negative	1 (reference)	0.580			1 (reference)	0.015		
	Positive	1.211 (0.615–2.385)				2.446 (1.188–5.039)			
Neural invasion	Negative	1 (reference)	0.148			1 (reference)	0.622		
	Positive	1.707 (0.827–3.521)				0.820 (0.374–1.802)			
Postoperative chemotherapy	Negative	1 (reference)	0.017	1 (reference)	0.006	1 (reference)	0.048		
	Positive	0.554 (0.341–0.899)		0.502 (0.307–0.820)		0.466 (0.218–0.994)			
Postoperative radiotherapy	Negative	1 (reference)	0.215			1 (reference)	0.060		
	Positive	0.602 (0.270–1.342)				0.453 (0.199–1.035)			
Postoperative endocrine therapy	Negative	1 (reference)	0.003	1 (reference)	0.000	1 (reference)	0.000	1 (reference)	0.000
	Positive	0.291 (0.129–0.655)		0.296 (0.177–0.497)		0.140 (0.062–0.319)		0.253 (0.145–0.441)	
Postoperative targeted therapy	Negative	1 (reference)	0.000	1 (reference)	0.000	1 (reference)	0.000	1 (reference)	0.000
	Positive	0.172 (0.085–0.347)		0.217 (0.137–0.345)		0.083 (0.037–0.188)		0.188 (0.119–0.295)	

^#^MPG, Miller–Payne grade; TLN, total lymph node; PLN, positive lymph node; ER, estrogen receptor; PR, progesterone receptor; HER2, human epidermal growth factor receptor 2; AR, androgen receptor; E-cad, E-cadherin; EGFR, epidermal growth factor receptor; TOP2A, topoisomerase II-α.

Moreover, the univariate analysis showed that LDH, dNLR, BIPI, PD-L1, neutrophils, lymphocyte, monocyte, tumor site, pathological N stage, pathological TNM stage, positive lymph node (PLN), PR, CK5/6, E-cad, epidermal growth factor receptor (EGFR), lymph vessel invasion (LVI), postoperative chemotherapy, postoperative endocrine therapy, and postoperative targeted therapy were associated with the prognosis of breast cancer patients for OS; however, the multivariate analysis found that only LDH, BIPI, PD-L1, neutrophils, PLN, PR, E-cad, postoperative endocrine therapy, and postoperative targeted therapy were the independent prognostic factors for OS (**Table 5**). Moreover, the results were displayed using forest plots and are shown in **Figure S2B**. BIPI was an independent prognostic factor for patients’ DFS and OS (DFS, hazard ratio (HR): 6.720, 95% confidence interval (CI): 1.629–27.717; OS, HR: 8.006, 95% CI: 1.638–39.119).

### Survival Analysis

**Figure 1:**
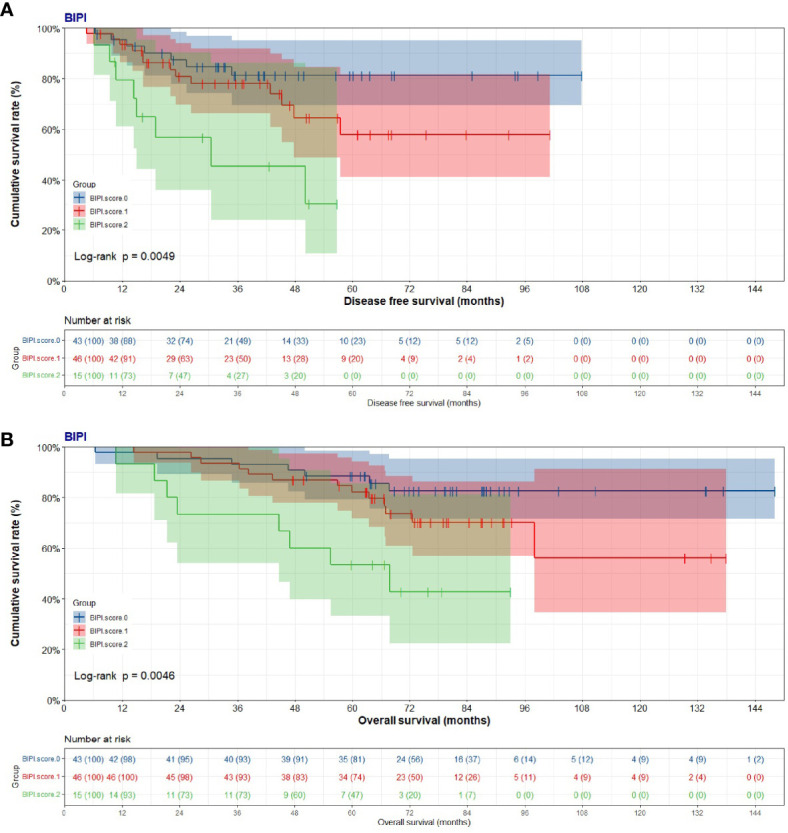
Kaplan–Meier curves for disease-free survival (DFS) and overall survival (OS). **(A)** Kaplan–Meier curves for DFS for breast immune prognostic index (BIPI). **(B)** Kaplan–Meier curves for OS for breast immune prognostic index (BIPI).

The mean DFS was 42.02 months (range from 6.33 to 107.77 months) in the BIPI score 0 group, 38.61 months (range from 4.67 to 101.30 months) in the BIPI score 1 group, and 26.01 months (range from 6.23 to 56.77 months) in the BIPI score 2 group, respectively. Overall, significant differences were found when comparing the three BIPI groups (p < 0.001). Moreover, the mean DFS of the BIPI score 0 group and BIPI score 1 group was significantly longer than that of the BIPI score 2 group (p = 0.007, and p = 0.025), respectively (**Figure 1A**). Furthermore, the mean OS was 77.61 months (range from 6.43 to 148.03 months) in the BIPI score 0 group, 71.83 months (range from 14.47 to 137.90 months) in the BIPI score 1 group, 53.15 months (range from 10.77 to 93.00 months) in the BIPI score 2 group, respectively. Analyses showed that there was a significant difference among the three groups compared (p < 0.001). Moreover, the mean OS of the BIPI score 0 group and BIPI score 1 group was significantly longer than that of the BIPI score 2 group (p = 0.011, and p = 0.041), respectively (**Figure 1B**).

According to the pretreatment optimal cutoff values of LDH and dNLR, we also analyzed the survival. A total of 83 cases (79.8%) had LDH <203.5 U/l, and 21 (20.2%) had LDH ≥203.5 U/l. Compared with the two groups, patients with low LDH had longer DFS and OS than those with high LDH (p = 0.018, and p = 0.011) (**Figure S3A**). A total of 49 cases (47.1%) had dNLR <1.67, and 55 cases (52.9%) had dNLR ≥1.67. Compared with the two groups, patients with low dNLR had longer DFS and OS than those with high dNLR (p = 0.039, and p = 0.043) (**Figure S3B**).

### Establishment and Validation of the Nomogram

**Figure 2:**
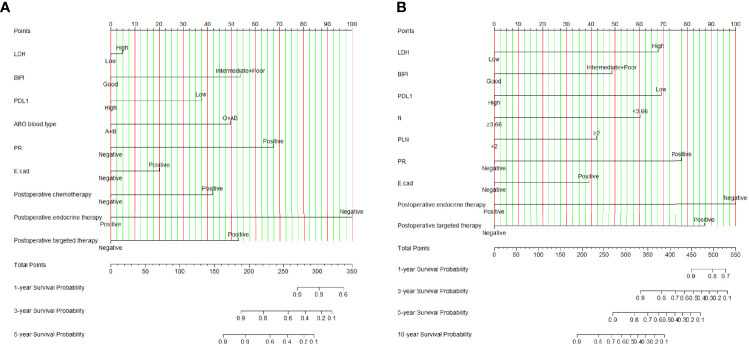
Breast immune prognostic index (BIPI)-based nomogram for predicting disease-free survival (DFS) and overall survival (OS). A straight upward line is drawn to determine the points for every predictor. The sum of these points is situated on the total points axis, and a straight downward line shows the 1-, 3-, and 5-year DFS estimated rates and 1-, 3-, 5-, and 10-year OS estimated rates. **(A)** BIPI-based nomogram for predicting disease-free survival (DFS). **(B)** BIPI-based nomogram for predicting and overall survival (OS). E-cad, E-cadherin; N, neutrophils; PLN, positive axillary lymph node.

According to the results of the univariate and multivariate Cox proportional hazard model, we constructed an effective and novel nomogram for the individualized assessment of DFS and OS after NACT and operation. In the nomogram, variables were imputed into weighted points, the sum of which was subsequently utilized to predict the 1-, 3-, and 5-year survival probabilities for DFS, and 1-, 3-, 5-, and 10-year survival probabilities for OS. A higher patient grade is associated with a lower survival probability. The nomogram for DFS had unique features, and integrated LDH, BIPI, PD-L1, ABO blood type, PR, E-cad, postoperative chemotherapy, postoperative endocrine therapy, and postoperative targeted therapy; it was generated as shown in **Figure 2A**. Moreover, the nomogram for OS had unique features, and integrated LDH, BIPI, PD-L1, neutrophils (N), PLN, PR, E-cad, postoperative endocrine therapy, and postoperative targeted therapy; it was generated as shown in **Figure 2B**. A nomogram with a C-index of 0.873 (95% CI: 0.779–0.966) and 0.801 (95% CI: 0.702–0.901) had a favorable performance for predicting DFS and OS survival rates for clinical use by combining immune scores with other clinical features. Moreover, we also conducted the dynamic nomogram, and the results are as shown in **Figures S4A, B**.

**Figure 3:**
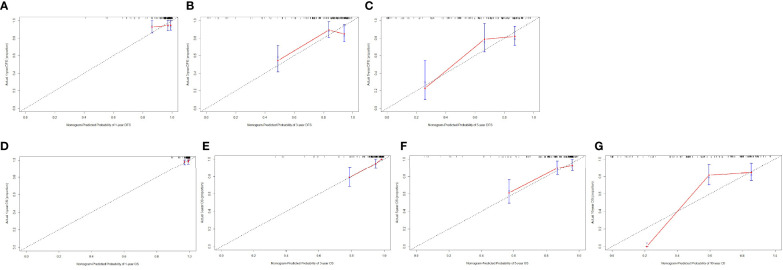
The calibration curves for predicting the 1-, 3-, and 5-year DFS rates and 1-, 3-, 5-, and 10-year OS rates. The X-axis presents the nomogram-predicted probability of disease-free survival (DFS) and overall survival (OS), and the Y-axis shows the actual DFS and OS. **(A)** The calibration curves for predicting the 1-year DFS rate in patients with breast cancer. **(B)** The calibration curves for predicting the 3-year DFS rate in patients with breast cancer. **(C)** The calibration curves for predicting the 5-year DFS rate in patients with breast cancer. **(D)** The calibration curves for predicting the 1-year OS rate in patients with breast cancer. **(E)** The calibration curves for predicting the 3-year OS rate in patients with breast cancer. **(F)** The calibration curves for predicting the 5-year OS rate in patients with breast cancer. **(G)** The calibration curves for predicting the 10-year OS rate in patients with breast cancer.

Furthermore, the calibration curves (1,000 bootstrap resamples) were used to assess the performance of the nomogram for the predicted and the actual probability of DFS and OS. The prediction line matched the reference line well for postoperative 1-, 3-, and 5-year survival DFS, which was an indication of good performance of the nomogram, especially for the 5-year DFS category (**Figures 3A–C**). Moreover, the prediction line matched the reference line well for postoperative 1-, 3-, 5-, and 10-year survival OS, showing good performance of the nomogram, especially in 3-year OS (**Figures 3D–F**). However, the prediction line matched the reference line not well for postoperative 10-year survival OS (**Figure 3G**).

### Comparison of Predictive Accuracy for DFS and OS Between Nomogram and BIPI by DCA

**Figure 4:**
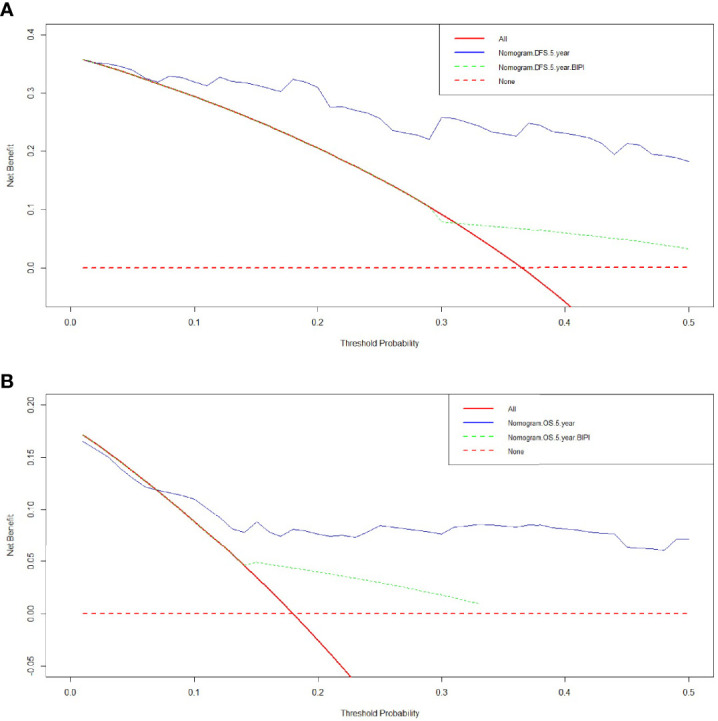
Decision curve analysis (DCA) of the nomogram and breast immune prognostic index (BIPI) for predicting the disease-free survival (DFS) and overall survival (OS). The X-axis represents the threshold probability, and the Y-axis shows the net benefit. The lines between the X-axis and the Y-axis display the benefit of different predictive variables. The red dotted line suggests that no patient has poor prognosis, while the red line indicates that all patients have poor prognosis. The blue line represents BIPI with other independent prognostic factors by the COX proportional hazard regression model, and the green line represents only BIPI. **(A)** DCA of the nomogram and BIPI for predicting the 5-year DFS. **(B)** DCA of the nomogram and BIPI for predicting the 5-year OS.

We used DCA to evaluate the clinical utility between the nomogram and BIPI by quantifying the net benefits at different threshold probabilities. A higher threshold probability represented better estimation for decision outcomes. Compared with BIPI, the nomogram model yielded the best net benefit across in the range of threshold probability for 5-year DFS and OS, indicating that its ability for clinical decision-making was better than only BIPI (**Figure 4**). The blue line represented BIPI with other independent prognostic factors by the COX proportional hazard regression model, and the green line represented only BIPI. Moreover, compared with the two lines, the blue line was obviously higher than the green line, which meant that the BIPI with other independent prognostic factors which were used to evaluate the prognosis showed better performance than only BIPI. Moreover, we also analyzed the clinical utility between BIPI and other factors (pathological N stage, pathological TNM stage) by DCA. The DCA demonstrated that the BIPI nomogram displayed better clinical predictive usefulness than the pathological N stage or pathological TNM stage alone (**Figure S5**).

## Discussion

This study aimed at investigating the potential prognostic significance of BIPI in breast cancer patients who received NACT to address research gaps in the literature. The present study demonstrated for the first time that BIPI was an independent prognostic factor by univariate and multivariate analyses for DFS and OS. According to the optimal cutoff values of LDH and dNLR, the BIPI was classified into three groups: BIPI score 0 (Good), BIPI score 1 (Intermediate), and BIPI score 2 (Poor). Overall, significant differences were found when comparing the three BIPI groups. Moreover, the mean DFS and OS of the BIPI score 0 group and BIPI score 1 group were significantly longer than those of the BIPI score 2 group. In order to more accurately predict the survival of breast cancer patients, we constructed a prognostic nomogram based on the independent prognostic factors by the Cox proportional hazard model. The nomogram predicts the 1-, 3-, and 5-year survival probabilities. Moreover, the prediction line matches the reference line well for postoperative survival DFS and OS survival by calibration curves, especially in 5-year DFS and 3-year OS. Furthermore, the nomogram model produced the best net benefit across in the range of threshold probability for 5-year DFS and OS than only by BIPI and could effectively provide the clinical decision-making for breast cancer.

Although the clinical application of multimodal treatments has been advancing, including surgery, chemotherapy, radiotherapy, and immunotherapy, the treatment and prognosis of advanced breast cancer are still not satisfactory (27). Hence, looking for the optimal individualized treatment and providing the appropriate prognostic indicators for breast cancer have become a research hotspot. In recent years, more attention has been paid to the immune response status in tumor cells, such as PD-1 and PD-L1, for mediating tumor progression and metastasis (28). It is well known that abnormal immune surveillance and immune escape of tumor cells play a critical role in affecting antitumor immune response and carcinogenesis (29, 30). Furthermore, the tumor immune microenvironment (TIME), including immune cells and inflammatory cells, influences the prognosis and effectiveness of treatment (31). Nevertheless, not all patients with a positive expression of PD-1 and PD-L1 can benefit from immunotherapy (32). Inflammation not only is of vital importance at different stages of tumor development and progression but also may have a negative or positive impact on tumor treatment response and immune monitoring (33, 34). It is also unknown whether combining BIPI and PD-L1 expression can lead to better prognoses for breast cancer patients. Therefore, accurate prognostic evaluation is an important prerequisite for the selection of appropriate treatments.

The BIPI score is composed of two values, namely, the levels of LDH and dNLR in the peripheral blood. The LDH is an enzyme responsible for the conversion of pyruvate to lactic acid during glycolysis, coded by two different genes LDH-A and LDH-B, and five isozymes (LDH1 to LDH5) with selective distribution among different tissues in serum are constructed (35, 36). LDH is a marker of inflammation, hemolysis, tissue injury, and myocardial infarction (37). Furthermore, it is elevated in many types of cancers as a potential diagnostic marker and has been linked to tumor growth, maintenance, and invasion (38). The dNLR has also been reported as a novel potential biomarker associated with different types of malignant tumors (39, 40). Moreover, a combination of the two parameters as an immune prognostic index (IPI)-based scoring system was used to evaluate the prognosis in various cancers, such as NSCLC (41). In Meyers’ study, the lung immune prognostic index (LIPI) correlates with survival outcomes in patients with NSCLC treated with immune checkpoint inhibitors (ICIs), and the intermediate and poor LIPI were independently prognostic of OS compared to good LIPI (42).

There are several plausible mechanisms to evaluate the relationship between BIPI and the prognosis of tumors. Hypoxia-inducible factor 1 (HIF-1) can be activated by the glycolytic metabolites, and it further upregulates angiogenic factors, leading to a feedforward stimulatory loop in cancer cells (43). Moreover, LDH is an enzyme involved in anaerobic glycolysis and gluconeogenesis, regulated by key oncogenic processes, such as phosphatidylinositol 3-kinase (PI3K), the target of rapamycin (TOR) kinase, and tumor hypoxia and necrosis (44). Hence, LDH is linked to angiogenesis and cancer progression, also depending on nutrient availability. Furthermore, LDH-A is very important in c-MYC-mediated cell transformation, and LDH-B is also critical in m-TOR-mediated tumorigenesis (45, 46). In recent clinical trials, serum LDH is a predictor of worse survival in diffuse large B-cell lymphoma (DBCL), advanced or metastatic breast cancer, and hepatocellular carcinoma (HCC) (47–49). Peripheral venous blood analysis can reveal the condition of the immune system. Available evidence has indicated that systemic inflammation is related to the prognosis of tumors and contributes to the pathogenesis and progression of cancers (50, 51). The dNLR was calculated by white blood cell and neutrophil. It is critical that neutrophil is the first line of human defense against infection and responds to different inflammatory signals (52). The neutrophil is an indicator of immune response and inflammatory and is involved in almost every stage of tumorigenesis and paradoxically shows antitumor and pro-tumor characteristics (53). The neutrophil also interacted with immune cells in the tumor microenvironment (TME) and peripheral blood (54). Furthermore, several studies also indicate that dNLR is an inflammation marker that can predict and reflect the prognosis of systematic inflammation in different types of tumors, for instance, non-colorectal gastrointestinal cancer and non-small cell lung cancer (NSCLC) (55, 56).

However, the present study had several limitations. First, this study is a retrospective study with a relatively small sample of breast cancer patients. Second, due to the presence of the eligibility criteria, the selection bias is difficult to eliminate. Third, while in line with the scope of the research question raised, some potential critical parameters associated with clinical prognosis have not been evaluated in the study, and the constructed nomogram was assessed by limited independent factors. Finally, as BIPI is a non-specific tumor marker, further study should consider further examining the correlation between BIPI and cancer prognosis in a prospective study.

## Conclusion

BIPI is found to be a significant prognostic factor and predictive biomarker for breast cancer patients. Patients with low immune scores are significantly related to better DFS and OS. Moreover, a novel nomogram based on immune scores may serve as a prognostic stratification tool to promote clinical decision-making.

## Data Availability Statement

The raw data supporting the conclusions of this article will be made available by the authors, without undue reservation.

## Ethics Statement

This study was approved by the ethics committee of Cancer Hospital Chinese Academy of Medical Sciences. The patients/participants provided their written informed consent to participate in this study.

## Author Contributions

Writing—original draft and writing—review and editing: LC, SH, and XK. Formal analysis: LC and XK. Data curation and investigation: LC and ZS. Methodology and supervision: YF and LZ. Resources, funding acquisition, and project administration: XL and JW. All authors contributed to the article and approved the submitted version.

## Funding

The work is partly supported by research grants from the National Nature Science Foundation of China (No. 81872160, No. 82072940, No. 82103047, No. 82102887, and No. 81802676), the Beijing Nature Science Foundation of China (No. 7191009, No. 7204293), the National Key R&D Program of China (No. 2018YFC1312100), the China National Key R&D (or Research and Development) Program (Nos. 2020AAA0105000 and 2020AAA0105004), the Special Research Fund for Central Universities, Peking Union Medical College (No. 3332019053), the Beijing Hope Run Special Fund of Cancer Foundation of China (No. LC2020L01, No. LC2019B03, No. LC2019L07), the Wuhan Youth Cadre Project (2017zqnlxr01 and 2017zqnlxr02), the Clinical Research Physician Program of Tongji Medical College, HUST (5001540018), the Golden Bridge Project Seed Fund of Beijing Association for Science and Technology (No. ZZ20004), the Chinese Young Breast Experts Research project (No. CYBER-2021-005), the 2021 Chaoyang District Social Development Science and Technology Plan Project (Medical and Health Field) (No. CYSF2115), the Beijing Xisike Clinical Oncology Research Foundation (No. Y-Young2021-0017), and the XianSheng Clinical Research Special Fund of China International Medical Foundation (No. Z-2014-06-2103).

## Conflict of Interest

The authors declare that the research was conducted in the absence of any commercial or financial relationships that could be construed as a potential conflict of interest.

The reviewer AL declared a shared affiliation with the authors LC, XK, YF, MZ, and JW at the time of review.

## Publisher’s Note

All claims expressed in this article are solely those of the authors and do not necessarily represent those of their affiliated organizations, or those of the publisher, the editors and the reviewers. Any product that may be evaluated in this article, or claim that may be made by its manufacturer, is not guaranteed or endorsed by the publisher.

## Supplementary Material

Supplementary Figure 1 Receiver operating characteristic curve (ROC) for LDH and dNLR.

Supplementary Figure 2 Forest plots for multivariate Cox proportional hazards regression model. A) Multivariate analysis for Disease free survival; 2) Multivariate analysis for Overall survival.

Supplementary Figure 3 Kaplan-Meier curves for disease-free survival (DFS) and overall survival (OS). **(A)** Kaplan-Meier curves for DFS for LDH; **(B)** Kaplan-Meier curves for OS for breast immune LDH; **(C)** Kaplan-Meier curves for DFS for dNLR; **(D)** Kaplan-Meier curves for OS for breast immune dNLR.

Supplementary Figure 4 Dynamic Nomogram for predicting the survival. **(A)** Dynamic Nomogram for DFS; **(B)** Dynamic Nomogram for OS.

Supplementary Figure 5 Decision curve analysis (DCA) of the BIPI and pathological N stage or pathological TNM stage for predicting the disease free survival (DFS) and overall survival (OS). The X axis represents threshold probability, and the Y axis shows net benefit. The lines between the X-axis and the Y-axis displays the benefit of different predictive variables. The red dotted line suggested that no patient has poor prognosis, while the red line indicated that all patients have poor prognosis. The blue line represented BIPI, and the green line represented only pathological N stage or pathological TNM stage. **(A)** DCA of the BIPI and pathological N stage for predicting the 5-year DFS; **(B)** DCA of the BIPI and pathological N stage for predicting the 5-year OS; **(C)** DCA of the BIPI and pathological TNM stage for predicting the 5-year DFS; **(D)** DCA of the BIPI and pathological TNM stage for predicting the 5-year OS.
