# Supplementary figures and images for: Preoperative Breast Immune Prognostic Index as Prognostic Factor Predicts the Clinical Outcomes of Breast Cancer Patients Receiving Neoadjuvant Chemotherapy
Source: Front Immunol. 2022 Mar 7;13:831848. doi: 10.3389/fimmu.2022.831848 (PMC8937039; doi:10.3389/fimmu.2022.831848)

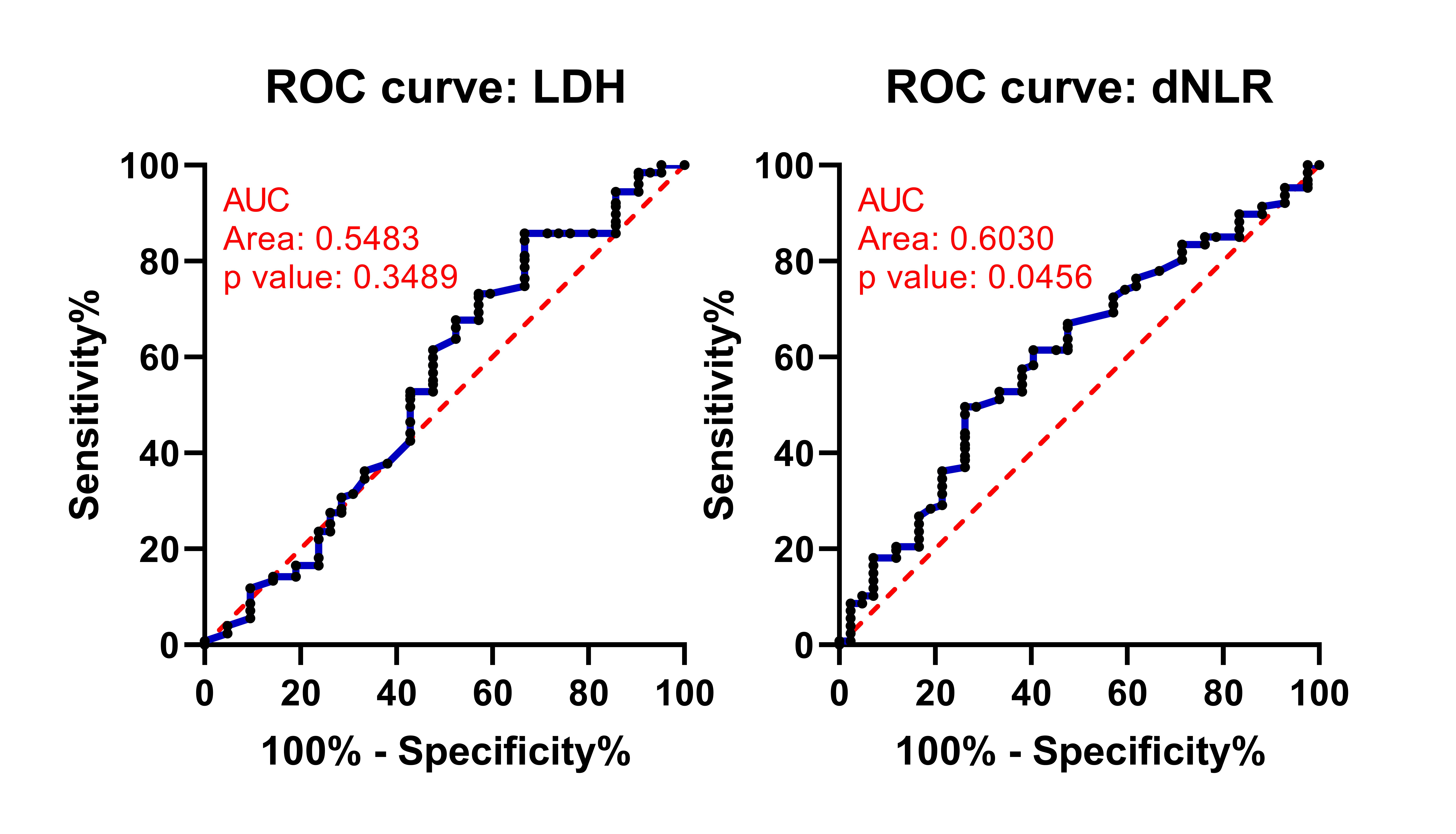

Supplement: Supplementary Figure 1 — Receiver operating characteristic curve (ROC) for LDH and dNLR. [file Image_1.jpg]

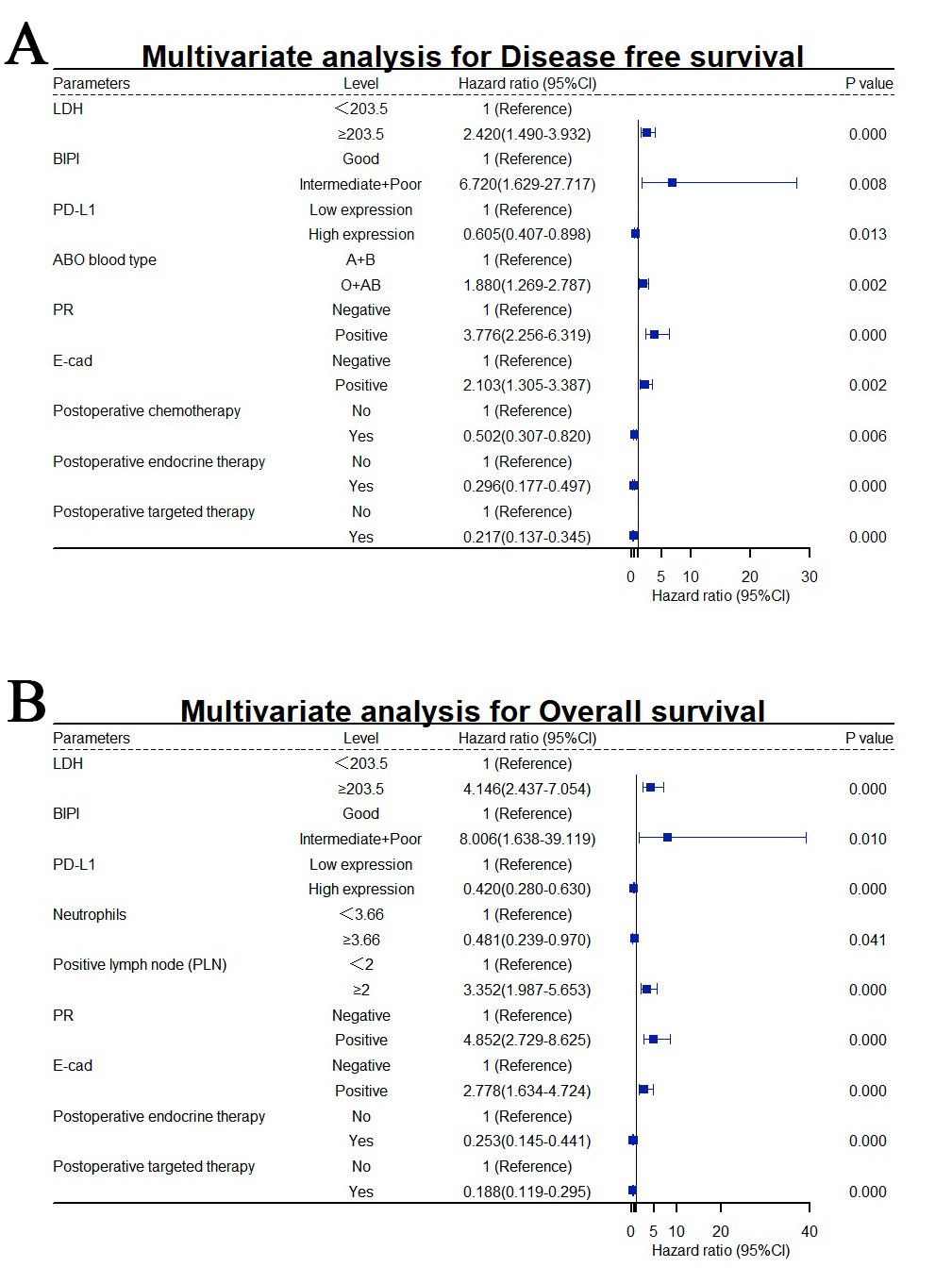

Supplement: Supplementary Figure 2 — Forest plots for multivariate Cox proportional hazards regression model. A) Multivariate analysis for Disease free survival; 2) Multivariate analysis for Overall survival. [file Image_2.jpg]

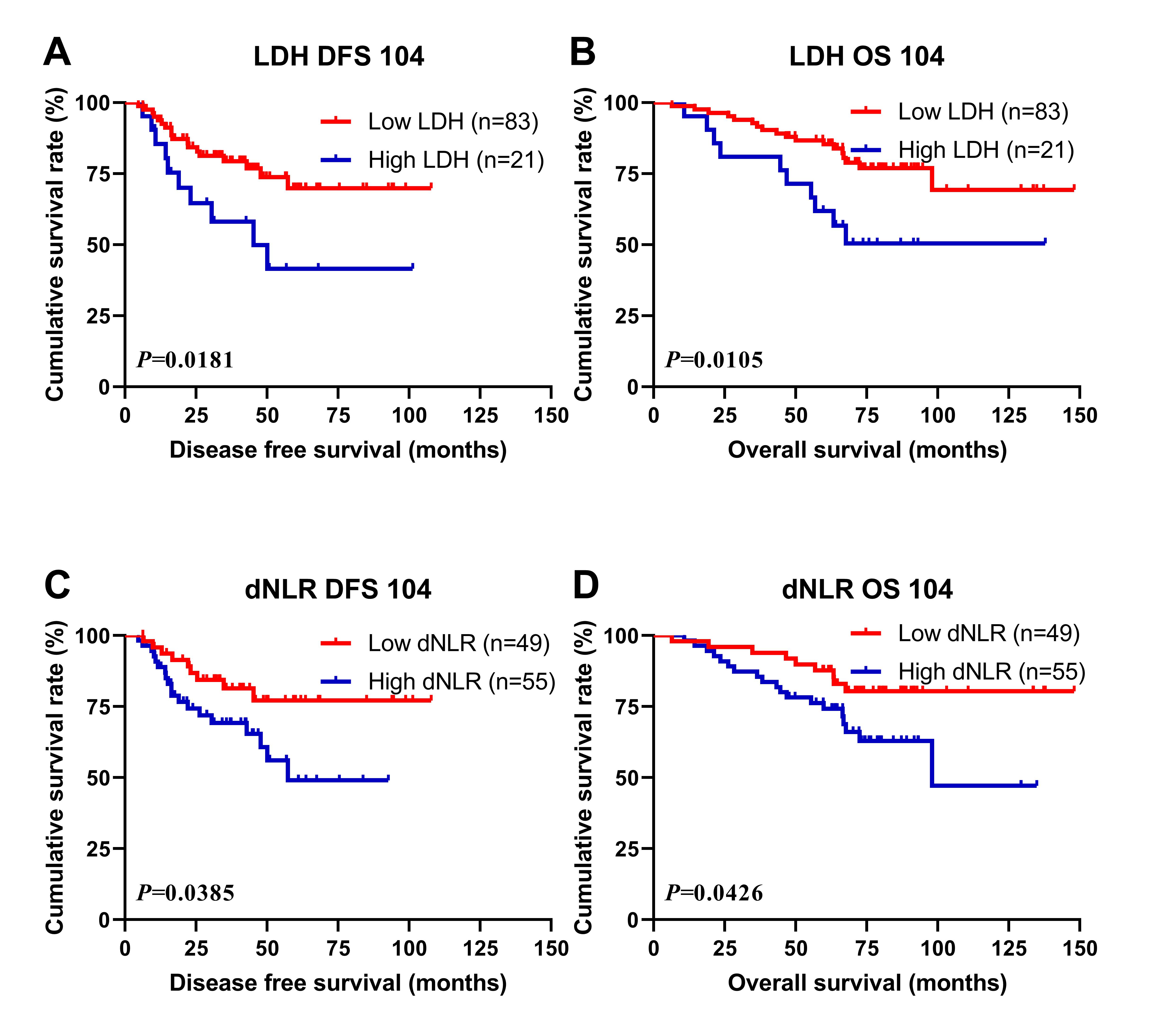

Supplement: Supplementary Figure 3 — Kaplan-Meier curves for disease-free survival (DFS) and overall survival (OS). (A) Kaplan-Meier curves for DFS for LDH; (B) Kaplan-Meier curves for OS for breast immune LDH; (C) Kaplan-Meier curves for DFS for dNLR; (D) Kaplan-Meier curves for OS for breast immune dNLR. [file Image_3.jpg]

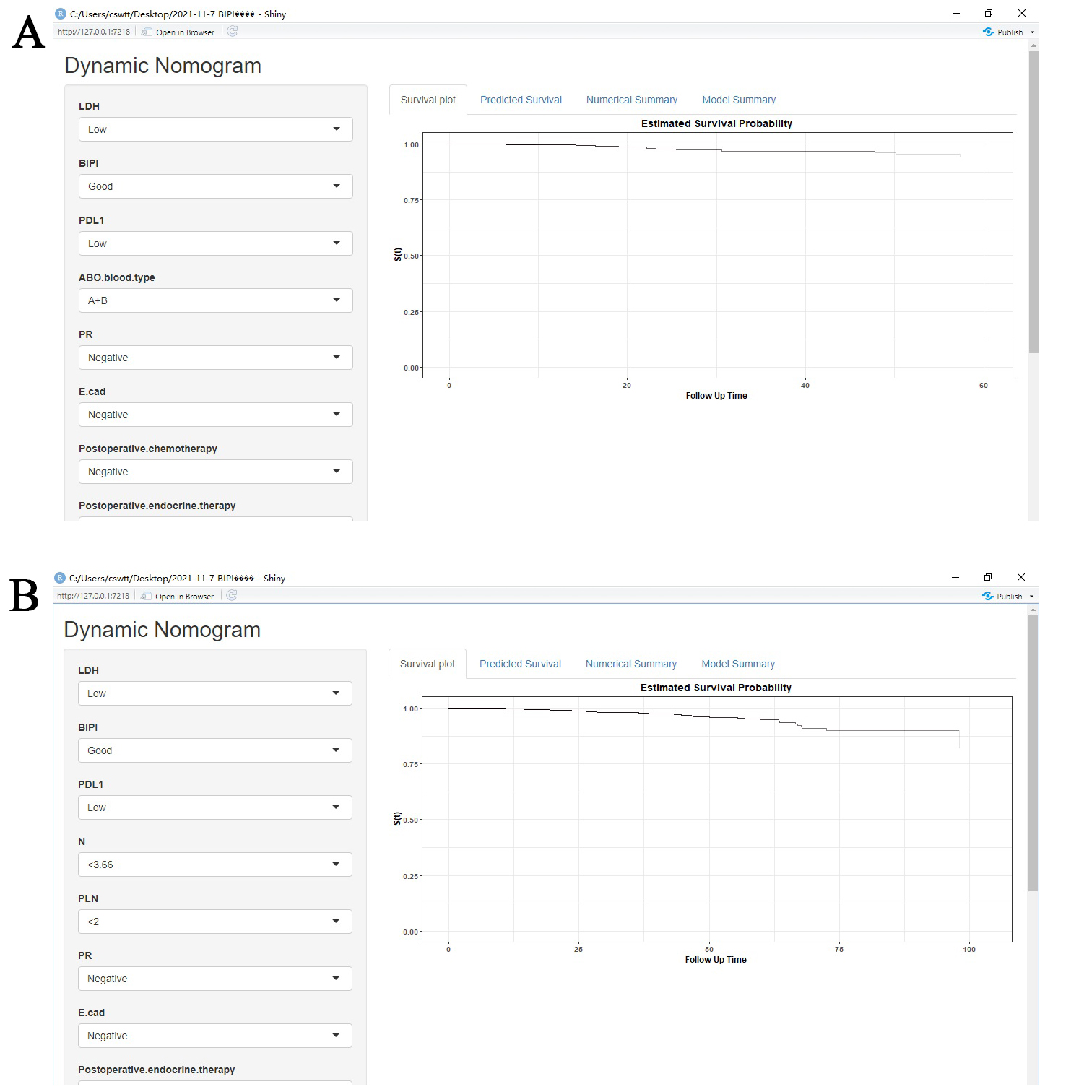

Supplement: Supplementary Figure 4 — Dynamic Nomogram for predicting the survival. (A) Dynamic Nomogram for DFS; (B) Dynamic Nomogram for OS. [file Image_4.jpg]

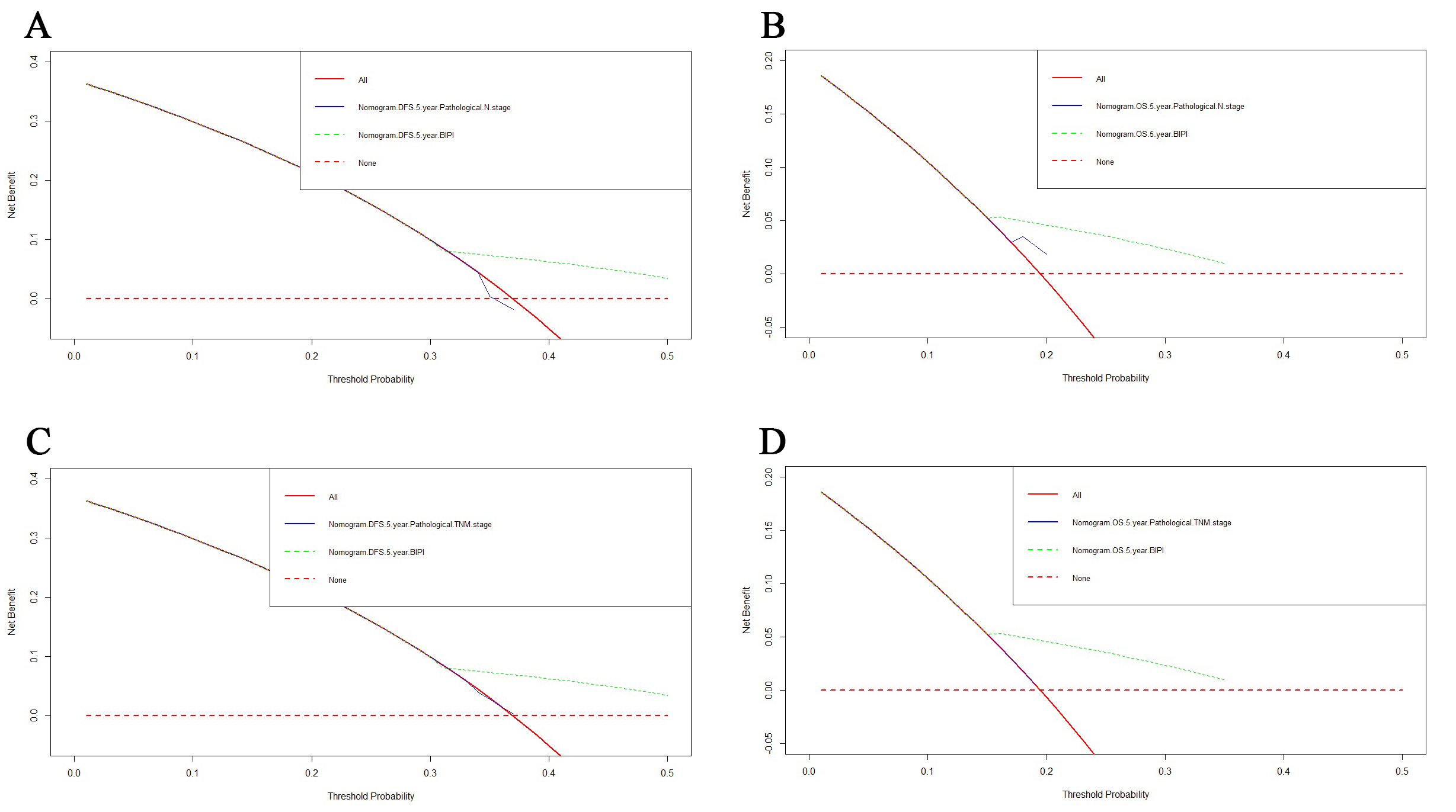

Supplement: Supplementary Figure 5 — Decision curve analysis (DCA) of the BIPI and pathological N stage or pathological TNM stage for predicting the disease free survival (DFS) and overall survival (OS). The X axis represents threshold probability, and the Y axis shows net benefit. The lines between the X-axis and the Y-axis displays the benefit of different predictive variables. The red dotted line suggested that no patient has poor prognosis, while the red line indicated that all patients have poor prognosis. The blue line represented BIPI, and the green line represented only pathological N stage or pathological TNM stage. (A) DCA of the BIPI and pathological N stage for predicting the 5-year DFS; (B) DCA of the BIPI and pathological N stage for predicting the 5-year OS; (C) DCA of the BIPI and pathological TNM stage for predicting the 5-year DFS; (D) DCA of the BIPI and pathological TNM stage for predicting the 5-year OS. [file Image_5.jpg]
